# Antibacterial and biocompatibility studies of triple antibiotics-impregnated external ventricular drainage: *In vitro* and *in vivo* evaluation

**DOI:** 10.1371/journal.pone.0280020

**Published:** 2023-01-05

**Authors:** Norased Nasongkla, Nattarat Wongsuwan, Aniroot Meemai, Adisorn Apasuthirat, Atthaporn Boongird

**Affiliations:** 1 Department of Biomedical Engineering, Faculty of Engineering, Mahidol University, Nakhon Pathom, Thailand; 2 Novatec Healthcare Company Limited, Samrong-Nua, Muang, Samutprakarn, Thailand; 3 Department of Surgery, Neurosurgical Unit, Faculty of Medicine, Ramathibodi Hospital, Mahidol University, Bangkok, Thailand; University of Sharjah, UNITED ARAB EMIRATES

## Abstract

Hydrocephalus is a neurological disease caused by an unusually high level of cerebrospinal fluid (CSF), which can be relieved by external ventricular drainage (EVD) insertion. However, the infection can lead to complications during the use of EVD. In this study, EVD was impregnated with three synergistic antibiotics, including rifampicin, clindamycin, and trimethoprim, to improve the antibacterial property. The impregnated drainage was studied for its characteristics *in vitro* and *in vivo*. Drug loading determination revealed that rifampicin had the highest concentration in the tube, followed by clindamycin and trimethoprim, respectively. *In vitro* cytotoxicity and hemolytic studies showed no toxic effects from antibiotics-impregnated EVD on fibroblast and red blood cells. For antibacterial testing, the impregnated EVD exhibited antibacterial activity against *Staphylococcus aureus* MRSA and *Staphylococcus epidermidis* up to 14 and 90 days, respectively. Moreover, biocompatibility and drug release into the bloodstream and surrounding tissues were investigated by implantation in rabbits for 30 days. Histology and morphology results showed that fibroblast cells began to adhere to the drainage surface and inflammatory cell numbers were noticeably small after the long-term implantation. In addition, there was no drug leakage to the bloodstream and surrounding tissues. Hence, this impregnated EVD can potentially be used for antibacterial application.

## 1. Introduction

Increased intracranial pressure (ICP) is a common neurological symptom from an unbalanced volume of contents in a cranial vault, consisting of the brain, cerebrospinal fluid (CSF), and blood. This disturbing event can notably cause some diseases such as hematoma, tumor, abscess, and hydrocephalus [1, 2]. Hydrocephalus is a neurological condition occurred by an unusually high level of cerebrospinal fluid (CSF), which results in its abnormal accumulation within the ventricles [3, 4]. The disease can occur at any age, and if it is left untreated, it may result in severe neurological injury and death [5]. Hydrocephalus can be treated long-term by insertion of a ventriculoperitoneal shunt (VP shunt) and short-term by external ventricular drainage (EVD) [3].

External ventricular drainage (EVD) is frequently used to release and monitor intraventricular pressure. There are some complications from EVD usage, including hemorrhage, dislodgement, and, especially, infection [6]. Neurological infection after surgery, so-called surgical site infections (SSIs), can lead to severe problems such as malfunction of the device, seizures and neurological deficits [3]. The infection rate associated with EVD is approximately 10% and significantly suggests a more extended stay at the hospital and resource consumption [2, 6]. Antibacterial EVDs are in great demand, while the problem of releasing antibacterials is still limited by the duration of antibacterial release and hinders their clinical applications. Antibacterial medical devices have been developed to reduce patients’ sickness, pain, cost, and time or improve patient life quality. There are various techniques to develop these devices, such as surface modification, layer-by-layer coating, dip coating, and spray coating [7–12]. For medical devices made of silicone, antibiotic impregnation is one of the techniques applied to this material.

This study aimed to develop antibacterial EVD impregnated with a synergistic ratio of rifampicin, clindamycin, and trimethoprim [13, 14] and evaluate its properties *in vitro* and *in vivo*. For *in vitro* studies, the impregnated EVD was evaluated for its drug loading, antibacterial activity against *S*. *aureus* (MRSA) and *S*. *epidermidis*, cytotoxicity, and hemolysis property. In addition, the loaded EVD was implanted into rabbits to investigate its biocompatibility, drug release in surrounding tissues, and morphology following the implantation. The studies were also compared with bare EVDs, which were used as a negative control.

## 2. Materials and methods

### 2.1 Materials

External ventricular drainages (EVD) were purchased from Medtronic, Inc. (Minnesota, USA). Medical grade clindamycin and rifampicin were purchased from Shaanxi Pioneer Biotech Co., Ltd (Xi’an, China). Medical grade trimethoprim was purchased from Hangzhou Royall Import & Export Co., Ltd (Zhejiang, China). Methicillin-resistant *Staphylococcus aureus* (MRSA, ATCC 43300), *Staphylococcus aureus* Xen 29 (ATCC 12600), *Staphylococcus epidermidis* (ATCC 12228), and mouse fibroblast cell line (L929) were purchased from American Type Culture Collection (ATCC) (Virginia, USA). All chemicals and solvents were purchased from Chemical Express Co., Ltd (Bangkok, Thailand). The test and control articles were sterized using ethylene oxide (EO) (Udomphan Supply Co., Ltd. -Nakhon Pathom, Thailand).

### 2.2 Animals

Healthy 3 to 4 months old, 6 New Zealand White rabbits of 2.8 to 3.2 kg body weight were divided into two groups (three animals for a control group and three animals for a study group). The control and study groups were implanted with unimpregnated and impregnated EVD, respectively. All animals were raised under 12 h:12 h of light and dark cycle at 20.0–22.0°C and 45.0–61.0% relative humidity. The rabbits were housed individually in hanging cages and fed with 8RD65 Lot 15. Guide for the care and use of laboratory animals (Institute of laboratory animal resources, National academic press 2011; NIH publication number #85–23, revised 2011) was followed strictly throughout the study.

### 2.3 Methods

#### 2.3.1 Impregnated EVD preparation

EVD was impregnated with drugs using a swelling technique. Three antibiotics, including rifampicin, clindamycin, and trimethoprim, were impregnated into the silicone tubes. In brief, rifampicin, clindamycin, and trimethoprim were all dissolved in chloroform at the ratio of 0.25:2:1. EVDs were immersed into the drug solutions for 2 hours while the mixture was stirred simultaneously. Next, EVDs were eluted with ethanol several times and left overnight to allow the ethanol to evaporate and the silicone structure to be restored. After that, the impregnated silicone was washed and sonicated twice in ethanol for 30 minutes each time. Finally, the loaded EVDs were rinsed with deionized water and allowed to be dried completely before storing.

#### 2.3.2 Drug qualification

The amount of impregnated antibiotics in silicone tubes was determined by high-performance liquid chromatography (HPLC) (Dionex Ultimate 3400, Thermo Fisher Scientific, USA). The stationary phase was a reverse phase C18 column (4.6 mm × 250 mm, 5 μm) (Zorbax Eclipse, Agilent Technologies, USA), while the mobile phase consisted of (A) phosphoric acid (0.01 M, pH 3) and (B) methanol. Furthermore, the determination was performed at the gradient ratio of time(min)/A(%)/B(%), which were 0.01/40/60, 1/40/60, 3/30/70, 5/0/100, 5.1/40/60, and 6/40/60, at the flow rate of 1 mL/min with 40°C of column temperature [15].

#### 2.3.3 *In vitro* study of impregnated EVD

Impregnated EVDs were determined for their drug loading content after the swelling. Firstly, the loaded drainage was submerged and sonicated in chloroform for 15 min. This process was repeated three times to confirm that it was sufficient to remove drugs from the silicone tube. The solvent was evaporated. Then, a methanol and water mixture (50:50) was added into all containers to dissolve the precipitated drugs. Then, the solutions were injected into HPLC, as described in **Section 2.3.2**. The experiment was performed in triplicate.

#### 2.3.4 Antibacterial testing

The antibacterial property of impregnated EVD was performed by Laboratory for Biocompatibility Testing of Medical Devices, Department of Biomedical Engineering, Faculty of Engineering, Mahidol University following CLSI M02 18^th^ edition and serial plate transferring test (SPTT) [16, 17]. The study was performed with methicillin-resistant *S*. *aureus* (MRSA) and *S*. *epidermidis*. Firstly, a bacterial inoculum was prepared and adjusted the concentration to 10^8^ CFU/mL by comparing to McFarland standard No. 0.5. Then, the bacteria were spread on Mueller-Hinton agar (MHA), and impregnated silicone tubes were plated in triplicate on the agar. The plate was incubated at 37°C overnight, and the diameters of the inhibition zone around the samples were measured. Furthermore, tubes were transferred to new inoculated agar daily until there were no inhibition zones around the loaded EVDs. For, the agar plate tested with *S*. *aureus* Xen 29 was incubated at 37°C overnight, and then it was observed under UVP ChemStudio Series (Analytikjena, Germany).

#### 2.3.5 Cytotoxicity and hemolysis testing

The cytotoxicity of the impregnated EVDs to fibroblast cells was performed by Laboratory for Biocompatibility Testing of Medical Devices, Department of Biomedical Engineering, Faculty of Engineering, Mahidol University according to ISO 10993-5(2009). Firstly, samples were extracted by submerging in MEM and incubated in an incubator shaker at 37°C for 24 hours. Simultaneously, L929 cells were cultured in a 96-well plate and incubated at 37°C for 24 hours. After that, cells were treated with various concentrations of extracted samples, and then the treated cells were incubated for 24 hours. Then, the culture media were removed, and MTT solutions (2 mg/mL in culture media) were added into each well. The plates were incubated at 37°C for 2 hours, and the solutions were removed. Finally, 100 μL of isopropanol was added into each well then, the absorbance of samples was measured at 570 nm using a microplate reader (Multiskan RC, Thermo Fisher, USA). The percentage of cell viability was calculated following Eq (1).

The hemolysis property of the impregnated EVD was performed by Laboratory for Biocompatibility Testing of Medical Devices, Department of Biomedical Engineering, Faculty of Engineering, Mahidol University followed ISO 10993–4 (2017). Briefly, the sample was immersed in PBS and incubated in the incubator shaker at 37°C for 24 hours. Then, the extracted solution was mixed with blood (1 mL), and the mixture was shaken at 37°C for 3 hours. Lastly, the solution was centrifuged and measured using a microplate reader for red blood cell concentration (Multiskan RC, Thermo Fisher, USA). The percentage of hemolysis was calculated following Eq (2).


$$\mathrm{Cell}\;\mathrm{viability}\;(\%)=\frac{\mathrm{Absorbance}\;\mathrm{of}\;\mathrm{cells}\;\mathrm{treated}\;\mathrm{with}\;\mathrm{sample}}{\mathrm{Absorbance}\;\mathrm{of}\;\mathrm{control}\;\mathrm{cells}}x\;100$$ (1)

$$\mathrm{Hemolysis}\;(\%)=\frac{\mathrm{Absorbance}\;\mathrm{of}\;\mathrm{RBC}\;\mathrm{treated}\;\mathrm{with}\;\mathrm{sample}}{\mathrm{Absorbance}\;\mathrm{of}\;\mathrm{control}\;\mathrm{RBC}}x\;100$$ (2)

#### 2.3.6 *In vivo* implantation

EVD implantation was performed in New Zealand White rabbits approved by National Laboratory Animal Center, Mahidol University, Thailand (Study number: non-GLP2020-31). Six rabbits were divided into two groups, which were implanted subcutaneously with control and impregnated EVDs in an abdominal area. For implantation operation, the rabbit was anesthetized through intramuscular injection of 5 mg/kg Xylazine. Subsequently, general anesthesia was maintained through automatic ventilator administration of a mixture of 2–4% isoflurane. The status of the anesthesia was carefully confirmed via the pupillary reflexes. The surgical rabbits were administrated with an analgesic (5 mg/kg Tramadol) reagent for five consecutive days after the surgery. The implantation was carried out for 30 days. During the implantation, all rabbits were observed, and collected their behavior, weight, feed, and water consumption dairy to monitor their health and unusual behaviors.

Moreover, blood from all rabbits was collected from their ear’s arteries 7 days and 24 hours before the surgery and 7, 14, 21, and 30 days after the surgery. The collected blood was centrifuged at 5,000 rpm, 10°C for 10 min to separate serum from the whole blood, then serums were kept at—80°C before the determination of drugs in serum. Drugs were extracted from the serums by protein precipitation technique. Briefly, serum was added with cold methanol then the mixture was centrifuged at 14,000 rpm, 10°C for 10 min. The supernatant was filtered and determined for drug concentration by HPLC [18].

After implantation, rabbits were sacrificed, and 5 x 5 cm of EVD surrounding tissues were collected and frozen. For animal sacrifice, animals were injected with 100 mg/kg of thiopental sodium. Drugs in tissues were extracted using a simple methanolic extraction [19] to determine drugs released from silicone tubes. Firstly, tissues were chopped into small pieces and submerged into methanol at the 3:1 ratio of methanol and tissues. The solution was homogenized by a probe homogenizer while it was immersed in ices. Next, the mixture was centrifuged at 4,000 rpm, 10°C for 5 min, and then the supernatant was again centrifuged at 14,000 rpm, 10°C for 10 min. The extracted solution was filtered and injected into HPLC.

#### 2.3.7 Histopathology

The histology of surrounding tissues was investigated after the rabbits were sacrificed. Briefly, collected tissues (1 cm from the EVD) were soaked in 10% formalin and cut into 5 μm using a microtome (RM2125 RTS, Leica, USA). After that, tissue pieces were stained with Meyer’s hematoxylin and eosin (H&E staining), then they were observed for the inflammatory cells under a bright-field inverted microscope (EP50, Olympus, Japan).

#### 2.3.8 Electron microscope

The morphology of the EVD before and after the implantation was examined under a scanning electron microscope (SEM; JEOL, JSM-6400, Japan). All EVD samples were coated with gold for 15 min then they were observed under SEM at 10 kV.

### 2.4 Statistical analysis

All the experiments were repeated at least three times and conducted in triplicate (n = 3), and error bars represented standard deviations (SD). Statistical analyses were performed using SPSS statistics. A comparison of obtained data for different samples was analyzed by one-way ANOVA. The significance level was set at P < 0.05.

## 3. Results and discussions

### 3.1 Characterization of drug-impregnated EVD

Triple antibiotic-impregnated EVDs were successfully prepared by swelling technique. The ratio of rifampicin, clindamycin, and trimethoprim was 0.25:2:1, respectively–which was the synergistic ratio to inhibit the bacteria [13, 14]. Loaded silicone tubes were investigated for drug loading content by HPLC. Results revealed that rifampicin had the highest concentration (650.48 ± 153.38 μg**/**cm^3^) in the silicone, while clindamycin (410.56 ± 2.50 μg**/**cm^3^) and trimethoprim (20.22 ± 0.56 μg**/**cm^3^) had lower concentration.

### 3.2 Antibacterial activity of impregnated EVD

**Fig 1:**
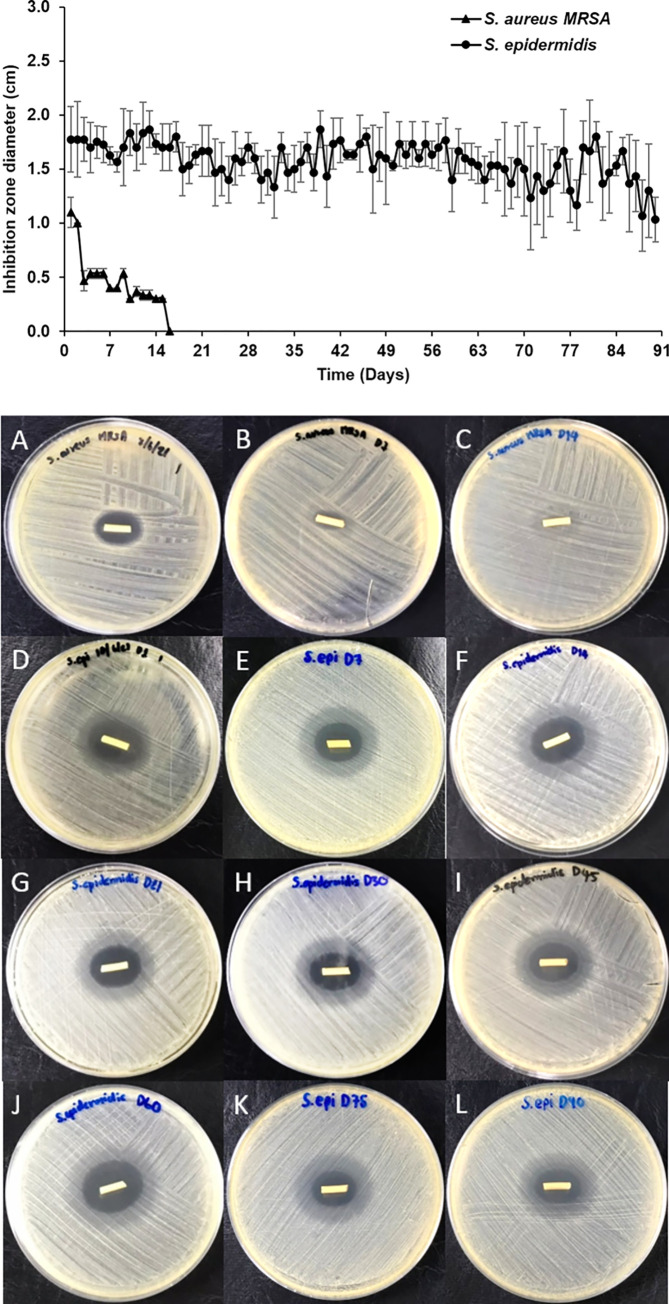
**A** Inhibition zone of impregnated EVDs against *S*. *aureus* MRSA and *S*. *epidermidis*. **B** Inhibition zone of impregnated EVDs against *S*. *aureus* MRSA after (A) 1, (B) 7, and (C) 14 days, and *S*. *epidermidis* after (D) 1, (E) 7, (F) 14, (G) 21, (H) 30, (I) 45, (J) 60, (K) 75, and (L) 90 days.

**Fig 2:**
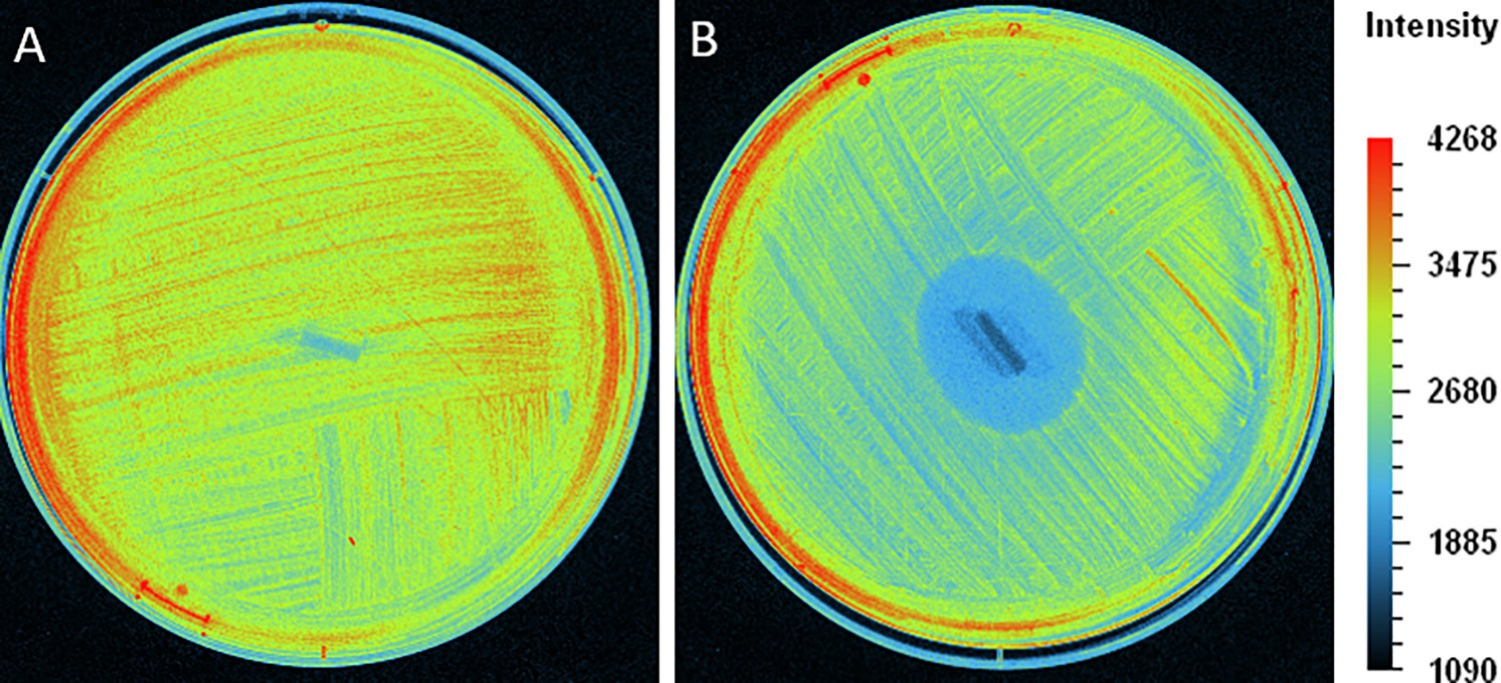
Inhibition zone of (A) bare and (B) impregnated EVDs against *S*. *aureus* Xen 29.

The antibacterial activity of impregnated EVDs was studied with *S*. *aureus* MRSA and *S*. *epidermidis* by agar diffusion assay as described in **Section 2.3.8**. Silicone tubes were transferred to a new agar plate every day, and the zone of inhibition was also measured daily. Results revealed that impregnated EVDs could inhibit the growth of *S*. *aureus* MRSA for 16 days, while it could inhibit the growth of *S*. *epidermidis for* at least 90 days (**Fig 1A and 1B**). Moreover, impregnated EVDs were examined for their antibacterial activity against *S*. *aureus* Xen 29, the bioluminescent bacteria. The bacteria were cultured and tested with the loaded silicone tubes and then observed under fluorescent light (UVP ChemStudio, Analytik Jena, USA). **Fig 2** shows the agar plates of bare and impregnated EVDs after culturing with *S*. *aureus* Xen 29 for one day. It should be noted that the bar indicates the bacterial intensity. Results represented the inhibition zone around the impregnated EVD, and the intensity was gradually increased from the zone to the border of the agar plate. Bare EVDs showed no inhibition zone, and the intensity was the same over the plate. It could be concluded that impregnated EVDs had the antibacterial property against *S*. *aureus* MRSA, *S*. *epidermidis*, and *S*. *aureus* Xen 29 compared to the bare EVDs.

### 3.3 *In vitro* study

**Fig 3:**
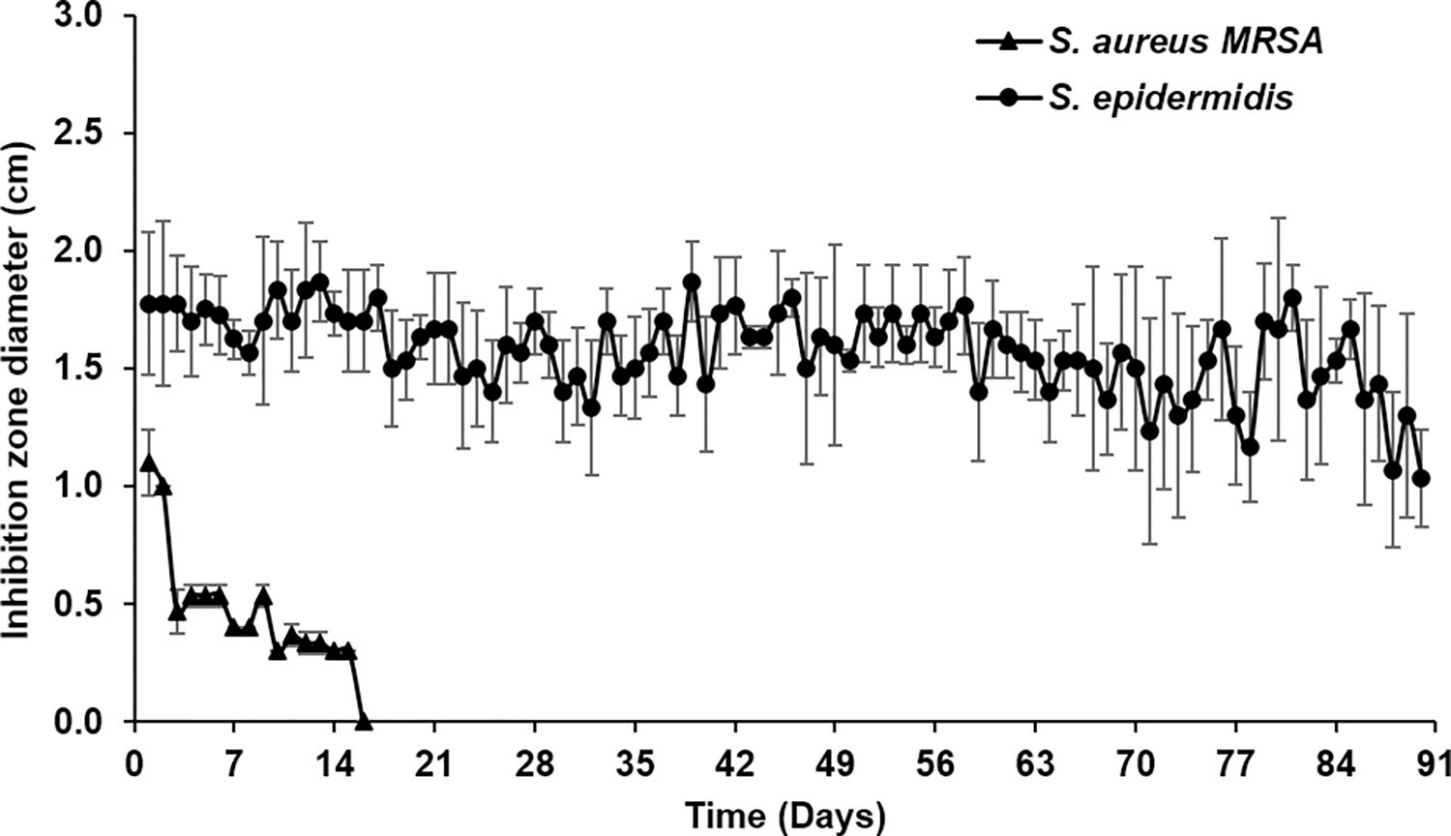
Cytotoxicity at different concentrations of extraction from impregnated EVD compared to the control group.

Impregnated EVDs were investigated for the cytotoxic effect on fibroblast cells (L929) and red blood cells (RBC), according to ISO 10993 and CLSI F756, respectively. From **Fig 3**, cell viability percentages of all concentrations of samples were over 80%. The results suggested that the impregnated silicone had no effect on the normal cells. The results suggested that the impregnated silicone had no effect on the normal cells.

Also, the impregnated silicone was tested with RBC to study the hemolytic effect. Hemolytic index could indicate the hemolytic results of samples after being incubated with blood. The hemolytic index of impregnated EVDs was 0.0, which indicated non-hemolysis of the EVD, while hemolytic indexes of positive and negative control were 103.7 and 0.0, respectively. It could be concluded that there was no effect from the EVD on red blood cells. Therefore, impregnated EVDs are biocompatible with fibroblast cells and RBC, and they are eligible to be studied further for *in vivo* study.

### 3.4 *In vivo* evaluation of impregnated EVDs

**Fig 4:**
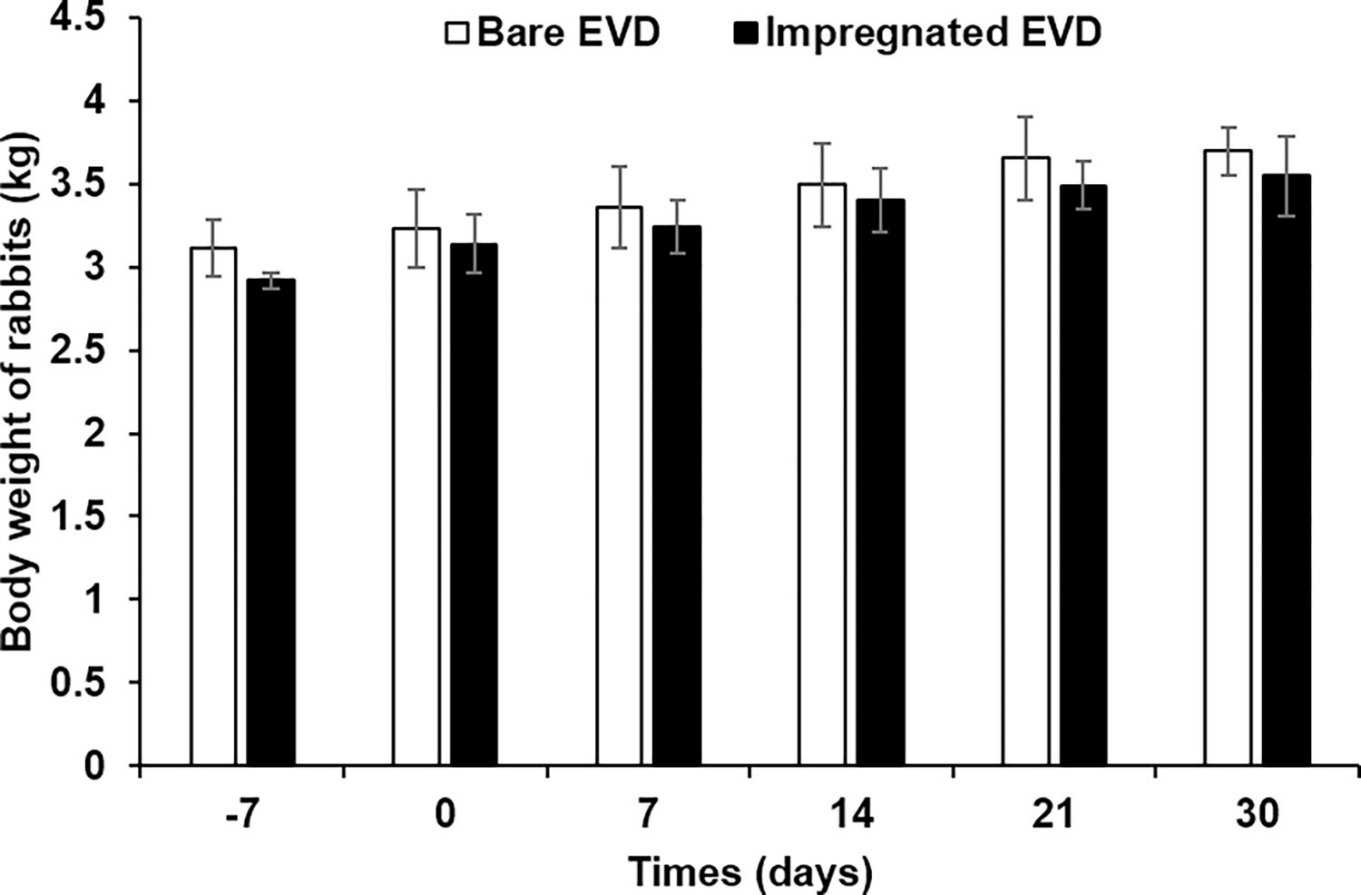
Body weight of rabbits after implantation with bare and impregnated EVD at different times.

New Zealand White rabbits were divided into two groups: a control group and a testing group. The control group was implanted with bare unimpregnated EVDs, while the testing group was implanted with impregnated EVDs. Animals were monitored for their health dairy during the implantation. **Fig 4** shows the average body weights of rabbits from both groups at different time intervals, which could be seen that their weight increased throughout the experiment. It should be noted that the *p-*value of impregnated EVD at day 0 and day 21 was below 0.05. Results from the observation of rabbit behaviors suggested that all rabbits were healthy and there were no unusual activities.

Furthermore, all rabbits’ blood was taken before and during the implantation to investigate drug release into the bloodstream. Drugs were separated from blood serum then drug concentrations were determined using HPLC. The results demonstrated that there was no drug release to the bloodstream compared to the control group, which was implanted with the bare EVD. Nevertheless, implant surrounding tissue from all rabbits was collected after implantation for 30 days. Tissues were digested using the methanolic extraction, and then drugs were determined using HPLC. The study revealed no drug release to the surrounding tissues compared to the tissue implanted with the bare silicone. Overall, all drugs were not released from the impregnated EVD into both bloodstream and surrounding tissues indicating the successful impregnation of antibiotics in the implants.

### 3.5 Histological analysis

**Fig 5:**
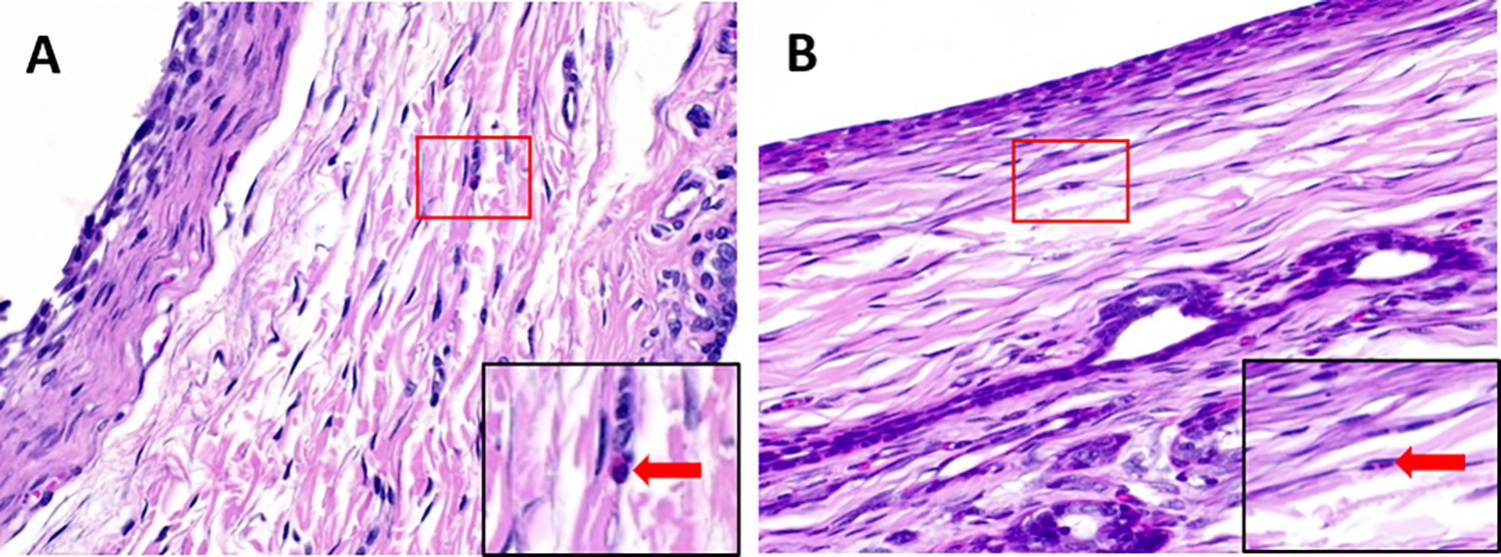
H&E staining of the surrounding tissue after implantation with (A) bare EVD and (B) impregnated EVD (Red arrow indicated the inflammatory cells).

Histopathology of EVD surrounding tissues was investigated to study inflammation from the EVD implantation as shown in **Fig 5**. Tissues from all rabbits were collected and dyed with H&E stain. The stained tissues were observed, and the inflammatory cells were counted under the microscope. The number of inflammatory cells in rabbit tissues implanted with bare EVD and impregnated EVD was found to be equal to 3.8 cells. It suggested that the bare and the impregnated EVD had the same property toward the surrounding tissue after implantation for 30 days.

### 3.6 Characterization of impregnated EVDs after implantation

**Fig 6:**
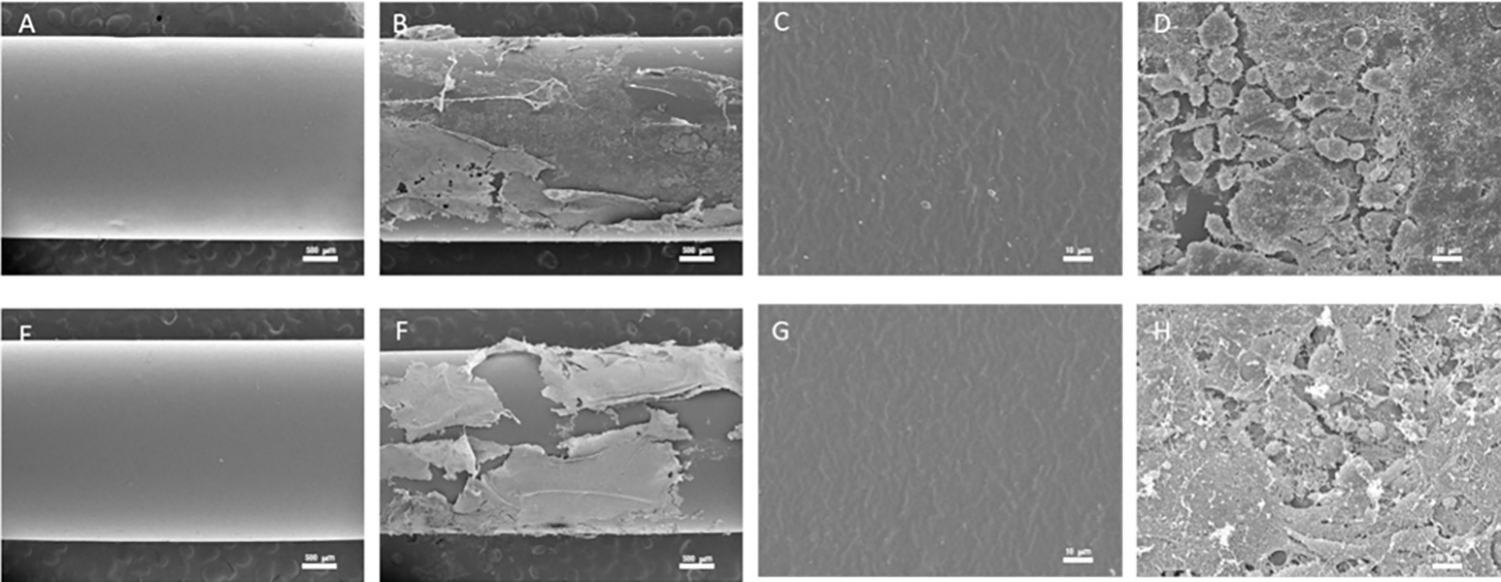
Morphology of EVDs before and after implantation. **A** and **E** represent bare and impregnated EVDs before implantation with 25x. **B** and **F** represent bare and impregnated EVDs after implantation with 25x. **C** and **G** represent bare and impregnated EVDs before implantation with 1000x. **D** and **H** represent bare and impregnated EVDs after implantation with 1000x.

Morphology of EVDs before and after implantation was observed under SEM, as shown in **Fig 6**. Before the implantation, both bare and impregnated EVD had the same morphology, which was a smooth and clear surface. In addition, there were layers of fibrous tissues on both bare and loaded EVD surfaces after implantation for four weeks. These fibrotic layers developed due to foreign body reactions that occurred during a normal wound healing process, supported by the histological results in **Section 3.5** of a small number of inflammatory cells (neutrophil and monocyte) in the implant surrounding tissues.

## 4. Conclusions

External ventricular drainage (EVD) was successfully impregnated with three types of antibiotics, including rifampicin, clindamycin, and trimethoprim. The impregnated silicone tubes showed long-term antibacterial property. For cytotoxicity and hemolysis testing, results indicated that there was no toxicity of the impregnated EVD toward fibroblast cells and red blood cells. Meanwhile, the animal study demonstrated that drugs in silicone tubes did not leak into the bloodstream and surrounding tissues. The histology and morphology revealed that the inflammatory cells were decreased while fibroblast cells started to adhere to the silicone surface after implantation for four weeks. For further study, the impregnated EVD will be tested for other biocompatibility evaluations, such as skin sensitization, skin irritation, acute systemic toxicity, subacute toxicity, and genotoxicity, before clinical studies and use in biomedical applications.
